# Glycemic control is associated with atrial structural remodeling in patients with type 2 diabetes

**DOI:** 10.1186/s12872-019-1249-2

**Published:** 2019-12-02

**Authors:** Qing Wang, Jing Wang, Pei Wang, Liaoyuan Wang, Lanting Jia, Xinyu Ling, Wang Xi, Jie Min, Hua Shen, Jian Xiao, Jinxiang Yuan, Zhinong Wang

**Affiliations:** 1 Center for Comprehensive Treatment of Atrial Fibrillation, Department of Cardiothoracic Surgery, Changzheng Hospital, Second Military Medical University, Shanghai, 20003 China; 2 Jining Medical University, Jining, 272067 Shandong China; 3 Department of Cardiothoracic Surgery, Shanghai Shuguang Hospital, Shanghai University of Traditional Chinese Medicine, Shanghai, 201203 China; 4 Hangzhou Sanatorium of Chinese People’s Liberation Army Air Force, Hangzhou, 310007 China; 5 Department of Ultrasound, Changzheng Hospital, Second Military Medical University, Shanghai, 200003 China

**Keywords:** Diabetes mellitus, Atrial fibrillation, Structural remodeling, Atrial fibrosis, Atrium enlargement, Echocardiography, Atrium enlargement, Echocardiography

## Abstract

**Background:**

Diabetes mellitus (DM) has been demonstrated to be a strong risk factor for development and perpetuation of atrial fibrillation (AF). However, how DM and glycemic control affect the pathogenesis of AF has not been sufficiently investigated, especially for the atrial structural remodeling.

**Methods:**

A total of 86 patients undergoing coronary artery bypass graft surgery were enrolled in this study, with atrium sample collected in the operation. The patients were divided into the DM group (*n* = 40) and the control group (*n* = 46) accordingly. Demographics, clinical data were collected and compared. Echocardiography, Masson staining and Western blotting were conducted to evaluate atrial structural remodeling.

**Results:**

There was no significant difference between the two groups in baseline characteristics (all *P* > 0.05). Fast blood glucose and HbA1c of DM group were significantly higher than the control group (*P* < 0.001). Echocardiography results demonstrated that the left atrium diameter (LAD) and left atrium volume index (LAVI) of DM group was significantly higher than the control group (*P* < 0.001). Masson staining showed that the collagen volume fraction (CVF), a quantitative indicator of fibrosis, was significantly higher in DM patients (*P* = 0.03). Western blot results indicated that the Collagen I of DM group was more expressed in the DM group than the control group (*P* < 0.001). Univariate linear regression revealed that the HbA1c level was significantly associated with both LAD (Y = 1.139X + 25.575, *P* < 0.001, R^2^ = 0.291) and CVF (Y = 0.444X + 29.648, *P* = 0.009, R^2^ = 0.078).

**Conclusions:**

DM was associated with atrial structural remodeling, including atrium enlargement and atrial fibrosis, which might be attributed to poor glycemic control.

## Background

Recent decades have witnessed a substantial increase in the prevalence of atrial fibrillation (AF), the most common sustained arrhythmia, which has caused great healthcare burden worldwide by leading to a higher risk of stroke and other complications [1–3]. Epidemiologic studies have identified several common risk factors for the development of AF, including chronic heart failure, male sex, coronary artery disease, hypertension, diabetes mellitus, left ventricular hypertrophy, age, obesity, smoking and alcohol [4–6]. Among them, diabetes mellitus (DM) was the most common chronic metabolic disease affecting about 400 million people every year, with an approximate incidence of 9% [7]e. It is estimated that compared with those without DM, patient with DM were subjected to a 40% higher risk of developing AF [8]. The validated relationship between DM and AF requires further studies to understand the elusive mechanism.

Numerous studies have demonstrated the fundamental process of atrial remodeling in the incidence, development, and perpetuation of AF, including the structural and electrical remodeling [9]. Previous studies have focused on oxidative stress, connexin remodeling, and glycemic fluctuations, and so on [10]. Echocardiography, considered as an essential tool to evaluate atrial structural remodeling, has been used widely to explore the effect of DM. However, the results are inconsistent, with different conclusions in the left atrium diameter of patients with DM [11, 12]. Atrial fibrosis, as another vital process associated with AF, has seldom been investigated before in DM patients, as well as its link with glycemic control. In this study, we compared the echocardiography and atrial fibrosis results between type 2 DM (T2DM) patients and controls; then, we evaluated their relationships with glycemic control.

## Methods

### Patients

From March 2016 to June 2018 in Changzheng Hospital affiliated with the Second Military Medical University, patients meeting the following criteria would be enrolled in this study. Inclusion criteria: 1.the patients must be diagnosed with coronary artery disease and planned to receive coronary artery bypass graft (CABG) surgery; 2. The patients and families must understand and agree to get enrolled in this experiment; 3. The patients and families must sign informed consent. Exclusion criteria: 1. patients who would receive off-pump CABG; 2. Patients who had structural heart disease, severe hepatic or renal dysfunction, metabolic syndrome, infectious disease, and cancer; 3. Patients age over 80 years old. After the enrollment, the patients were divided into the DM group and the control group according to their conditions with or without type 2 DM (T2DM). The diagnosis of T2DM was mostly based on medical history. The newly diagnosed T2DM patients were confirmed with diagnostic criteria by the American Diabetes Association (ADA) [13].

**Fig. 1:**
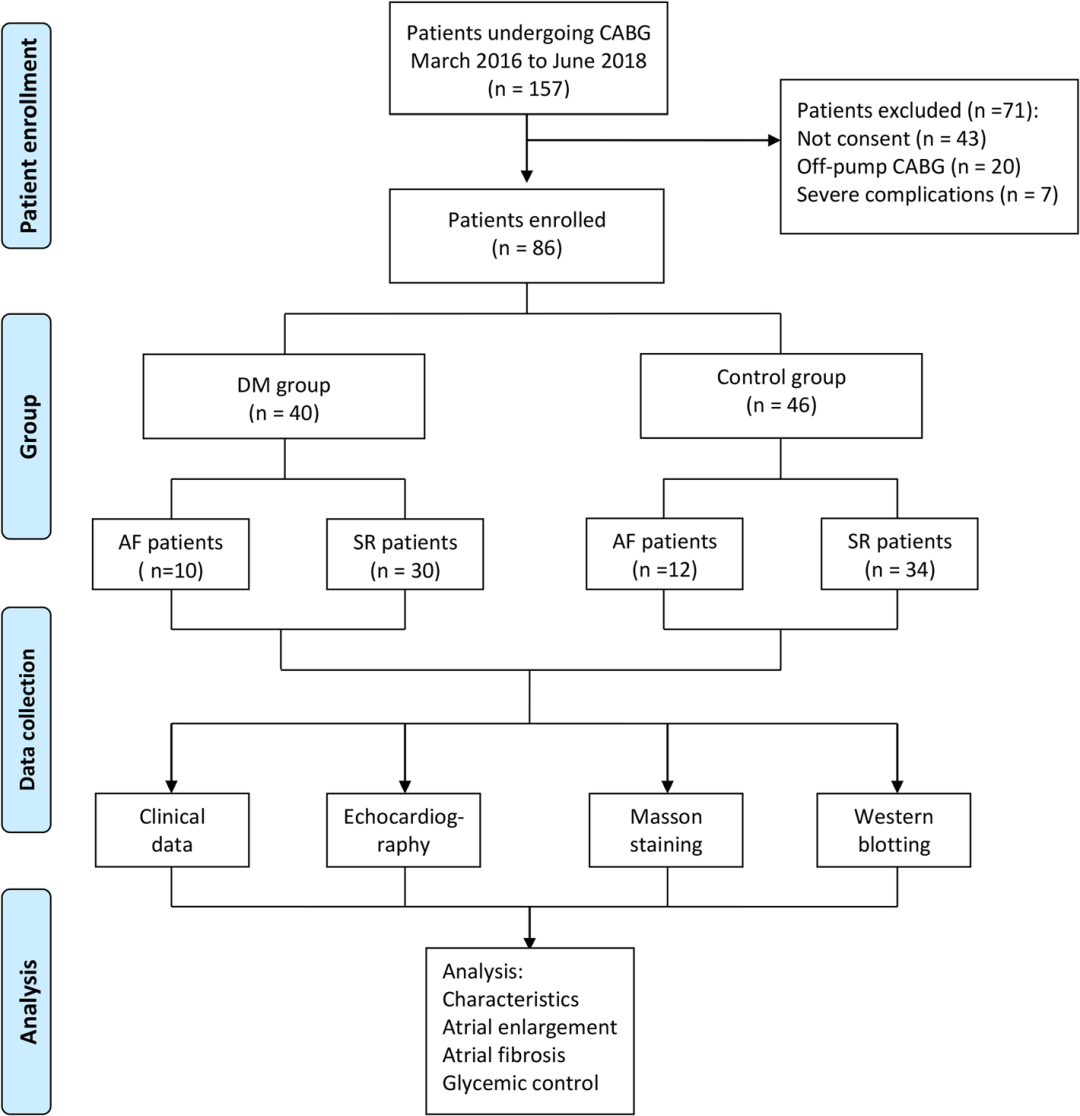
Flowchart of the study design

Demographical data, baseline characteristics, and clinical data of both groups were collected for analysis. The study design was shown in the flow chart of Fig. 1. This study was approved by the Committee on Ethics of Biomedicine of the Second Military Medical University. This study also complied with the Declaration of Helsinki, and signed, written informed consent was obtained from all subjects included in this study.

### Transthoracic echocardiography

All patients were given transthoracic echocardiography (TTE) with a 3.5-MHz transducer (Vivid 9, GE-Vingmed Ultrasound AS; Horten, Norway) before the surgery. The criteria set by the American Society of Echocardiography guidelines was obeyed during the examination, and the performer was blinded to the clinical details and personal information. Routine measurements of left atrium diameter (LAD) and left ventricular ejection fraction (LVEF) were performed. LAD referred to the distance from the leading edge of the posterior aortic wall to the leading side of the posterior LA wall in end-systole at the parasternal long-axis view, while the LVEF was calculated by Simpson method. LA volume was indexed to the body surface area.

As previously described, the pulse-wave Doppler velocity was recorded from the apical four-chamber view, and the Doppler sample was placed between the tips of the mitral valve leaflets. Influx blood from the mitral valve was used to measure early (E) and late (A) amplitude, E / A ratio, E deceleration time (DT) and isovolumic relaxation time (IVRT), isovolumetric contraction time (IVCT) and ejection time (ET) [14].

### Sample acquisition

During the CABG operation, the atrial myocardium (average 0.1 g) from the right atrial appendage tissue was acquired before suture of the right atrium and withdraw of cardiopulmonary bypass. Then the collected atrium tissue was divided into two portions, one was frozen immediately at − 80 °C for protein denaturation and western blot, and the other was immersed in neutralized formalin for Masson staining.

### Masson staining and Western blotting

As we previously reported [15], the Masson triple staining and Western Blotting were done according to the protocols. For Masson staining, the sections were observed under a light microscope, and the image data were collected to calculate the volume fraction of collagen (CVF% = average collagen area/area of total field× 100).

For Western Blotting, the protein expression level of Collagen I and Collagen III were tested, with the antibodies including anti-collagen I (Boster Biological Technology, Wuhan, China) and anti-collagen III (Boster Biological Technology, Wuhan, China) adopted, and anti-GAPDH used as an internal control. Image J software was used to calculate the relative optical density of the bands.

### Statistical analysis

SPSS 22.0 software (IBM, Almonte, NY, USA) was used for statistical analysis. Continuous data, when distributed normally, were expressed as mean ± standard deviation, while the categorical data were expressed as percentages.

Comparison of baseline characteristics, demographical data, and clinical data was conducted by Student-t test or Chi-square test depending on the variable category. Other variables including the echocardiography, the CVF and relative optical density of Western blotting were compared between groups by Student-t test. A linear regression model was adopted to investigate the relationship between HbA1c and LAD, as well as CVF. Multivariate linear regression analysis between LAD, CVF and other common risk factors of age, sex, smoking, BMI, hypertension, HbA1c was conducted. A *P* value of less than 0.05 was considered as statistical significant.

## Results

### Demographics and baseline characteristics

The demographics and baseline characteristics of both groups were shown in Table 1. As for demographics, there was no significant difference between the two groups in age, sex, BMI, and smoking status (all *P* > 0.05). There was also no statistical difference in NYHA grade and other comorbidities (all *P* > 0.05). Notably, the AF proportion was also similar between the two groups (*P* = 0.908). In terms of laboratory results, other than fast blood glucose (FBG) and HbA1c, which was significantly higher in DM group (*P* < 0.001), there was no statistical difference in other indicators, including hemoglobin (*P* = 0.093), creatinine (*P* = 0.458), and LDL-C (*P* = 0.610). No statistical difference was found in drug use between two groups(all *P* > 0.05).

**Table 1:** Demographical and clinical data of the DM group and the control group

Variables	DM group (*n* = 40)	Control group (*n* = 46)	t/χ^2^	P
Demographics				
Age(y)	61.5 ± 7.8	58.8 ± 8.3	1.519	0.133
Sex(male%)	25 (62.5%)	31 (67.4%)	0.225	0.635
BMI(kg/m^−2^)	22.1 ± 3.3	21.5 ± 3.8	0.789	0.433
Smoking	11 (27.5%)	10 (21.7%)	0.385	0.535
NYHA functional class			3.863	0.277
I	5 (12.5%)	13 (28.3%)		
II	15 (37.5%)	17 (37.0%)		
III	10 (25.0%)	9 (19.6%)		
IV	10 (25.0%)	7 (15.2%)		
Comorbidities				
Hypertension	9 (22.5%)	17 (37.0%)	2.120	0.145
Atrial fibrillation	10 (25.0%)	12 (26.1%)	0.013	0.908
Stroke	2 (5.0%)	1 (2.2%)	0.508	0.476
COPD	3 (7.5%)	8 (17.4%)	1.877	0.171
Laboratory results				
Hemoglobin (g/dL)	121.1 ± 23.7	112.5 ± 23.2	1.699	0.093
Creatinine (mg/dL)	0.725 ± 0.182	0.752 ± 0.156	0.745	0.458
LDL-C (mg/dL)	137.0 ± 17.5	139.0 ± 17.6	0.512	0.610
FBG (mg/dL)	193.5 ± 60.6	106.7 ± 20.6	9.134	< 0.001
HbA1c (%)	8.5 ± 2.3	5.3 ± 0.9	8.559	< 0.001
Drug use				
Aspirin	25 (62.5%)	30 (65.2%)	0.069	0.794
Nitrates	38 (95.0%)	40 (87.0%)	1.641	0.200
ACEI/ARB	5 (12.5%)	11 (23.9%)	1.840	0.175
Statins	21 (52.5%)	18 (39.1%)	1.543	0.214
β-blockers	12 (30.0%)	20 (43.5%)	1.664	0.197
Calcium channel blockers	3 (7.5%)	6 (13.0%)	0.702	0.402

BMI, Body mass index; NYHA, New York Heart Association; COPD, Chronic obstructive pulmonary disease; LDL-C, Low-density lipoprotein cholesterol; FBG, Fast blood glucose; HbA1c, hemoglobin A1c

### Transthoracic echocardiography

**Fig. 2:**
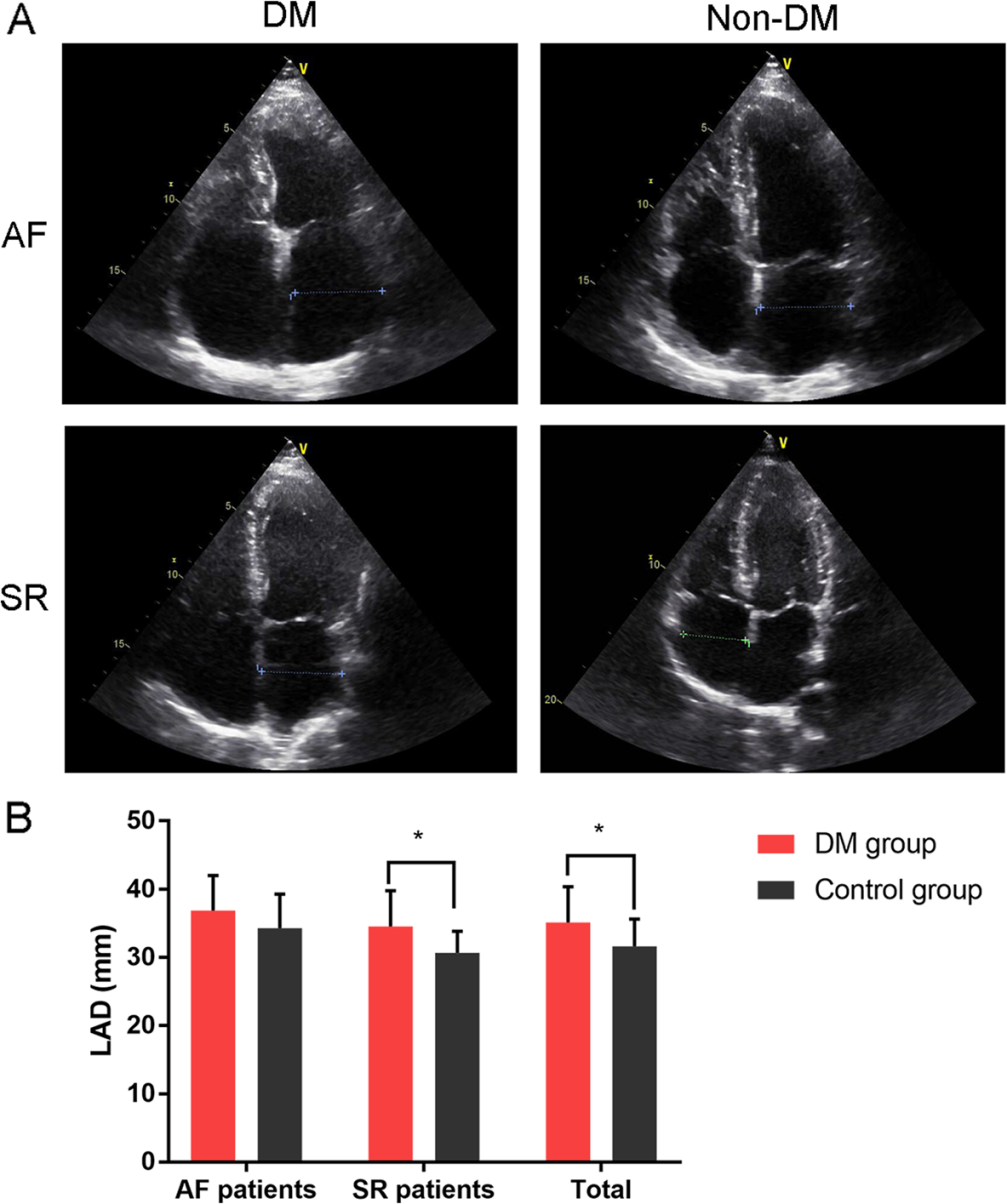
Echocardiography results of DM group (*n* = 40) and control group (*n* = 46). **a**) Representative sections in 4-chamber (4Ch) apical views; **b**) Comparison of LAD between DM group and control group. *P = 0.001. DM, diabetes mellitus; LAD, left atrium diameter

The TTE data was shown in Table 2. We conducted cross-sectional echocardiography and pulse-wave Doppler. Cross-sectional echocardiography showed that the interventricular septum thickness (IVST) of the DM group were significantly higher than those of the control group (*P* = 0.005). The representative echocardiography pictures were shown in Fig. 2, which demonstrated that LAD, and left atrium volume index (LAVI) of the DM group was significantly higher than the control group (*P* = 0.001). Doppler echocardiography results presented that E/A ratio of the DM group was also significantly lower than that of the control group (*P* = 0.025).

**Table 2:** Cross-sectional echocardiography and Doppler results of the DM group and the control group

Variables	DM group (*n* = 40)	Control group (*n* = 46)	t	P
Cross-sectional echocardiography				
LVEDD (mm)	46.4 ± 4.5	44.7 ± 5.0	1.625	0.108
LVESD (mm)	28.4 ± 2.5	27.5 ± 2.6	1.672	0.098
IVST (mm)	9.75 ± 1.28	8.87 ± 1.55	2.859	0.005
PWT (mm)	9.90 ± 1.11	9.57 ± 1.18	1.330	0.187
LVMI (g/m^2^)	90.0 ± 21.6	93.4 ± 28.2	0.616	0.540
Aortic diameter (mm)	27.5 ± 3.8	28.8 ± 2.9	1.750	0.084
LAD (mm)	35.1 ± 5.3	31.6 ± 4.0	3.494	0.001
LAVI (mL/m^2^)	31.1 ± 4.3	28.4 ± 2.7	3.566	0.001
LVEF (%)	51.9 ± 10.6	54.2 ± 10.7	1.027	0.307
PAP systolic (mmHg)	30.5 ± 5.1	31.7 ± 4.6	1.152	0.252
Doppler parameters				
Mitral E velocity (cm/s)	82.6 ± 15.9	84.9 ± 13.5	0.707	0.481
Mitral A velocity (cm/s)	72.7 ± 15.9	75.3 ± 20.5	0.656	0.513
E/A ratio	1.09 ± 0.29	1.26 ± 0.39	2.285	0.025
DT (ms)	182.1 ± 27.1	179.6 ± 24.3	0.452	0.652
IVRT (ms)	95.2 ± 12.0	95.0 ± 12.0	0.110	0.913

LVEDD, left ventricular end diastolic diameter; LVESD, left ventricular end-systolic diameter; IVST, interventricular septum thickness; PWT, posterior wall thickness; LVMI, left ventricular mass index; LAD, left atrium diameter; LAVI, left atrium volume index; LVEF, left ventricular ejection fraction; PAP, pulmonary artery pressure; DT, mitral E-wave deceleration time; IVRT, isovolumetric relaxation time

We also conducted a subgroup comparison of AF patients and sinus rhythm (SR) patients. For AF patients, although the LAD of the DM group was higher, the difference was not significant (*P* = 0.250). However, for SR patients, the difference of LAD between DM group and the control group was substantial (*P* = 0.001).

### Atrial fibrosis

**Fig. 3:**
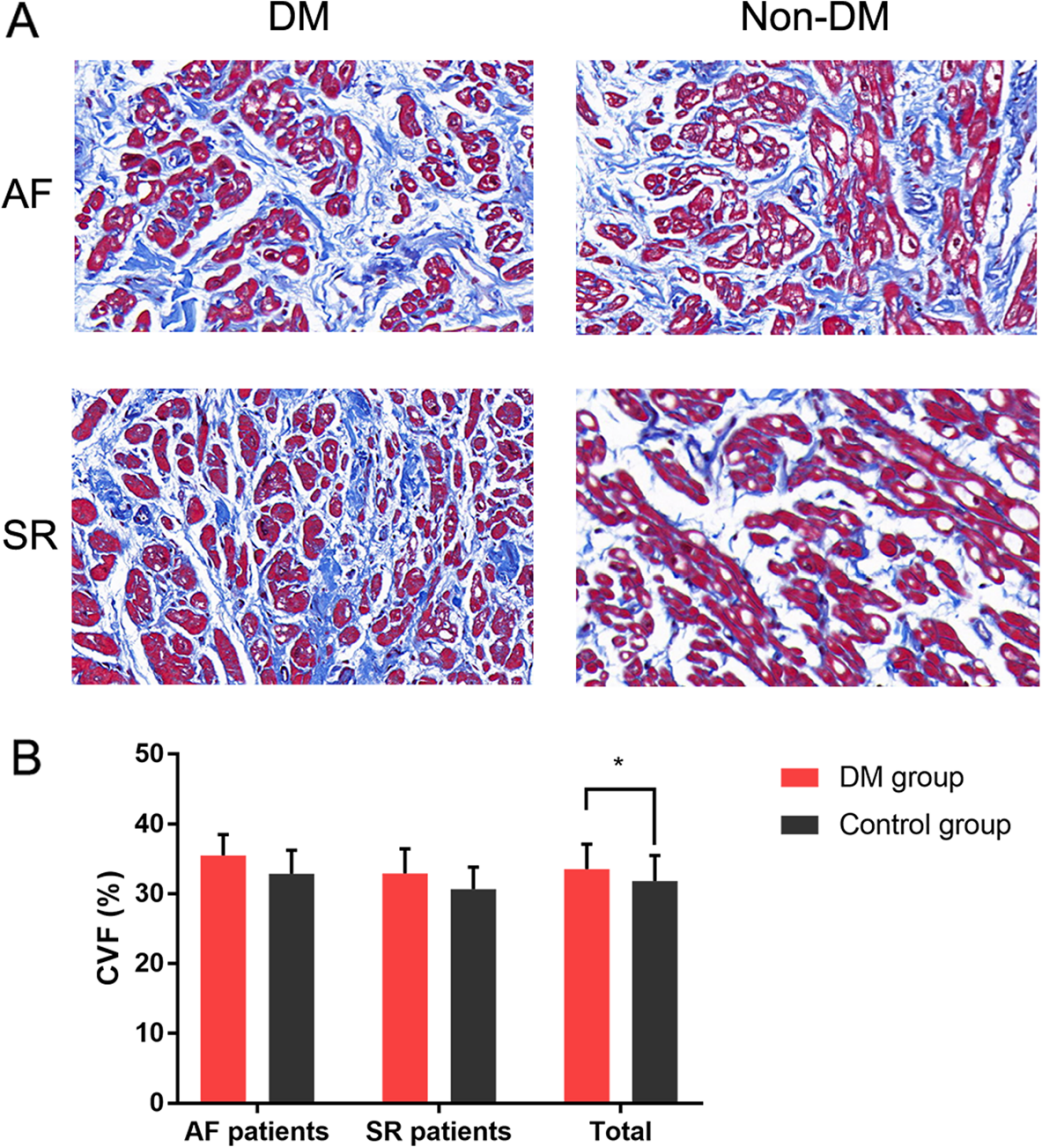
Masson staining and quantitative results of DM group (n = 40) and control group (n = 46). **a**) Representative sections of Masson staining; **b**) Quantitative results of Masson staining. * *P* = 0.03

Masson triple staining was used to examine the fibrosis extent of the atrium tissue, shown in Fig. 3. Representative sections of AF patients in the DM group, SR patients in the DM group, AF patients in the control group, and AF patients in the control group were presented in Fig. 3**a**. Although there was no significant difference in CVF between DM group and control group in AF subgroup (*P* = 0.075) and SR subgroup (*P* = 0.113), the CVF of DM group was significantly higher than the control group in total (*P* = 0.03).

**Fig. 4:**
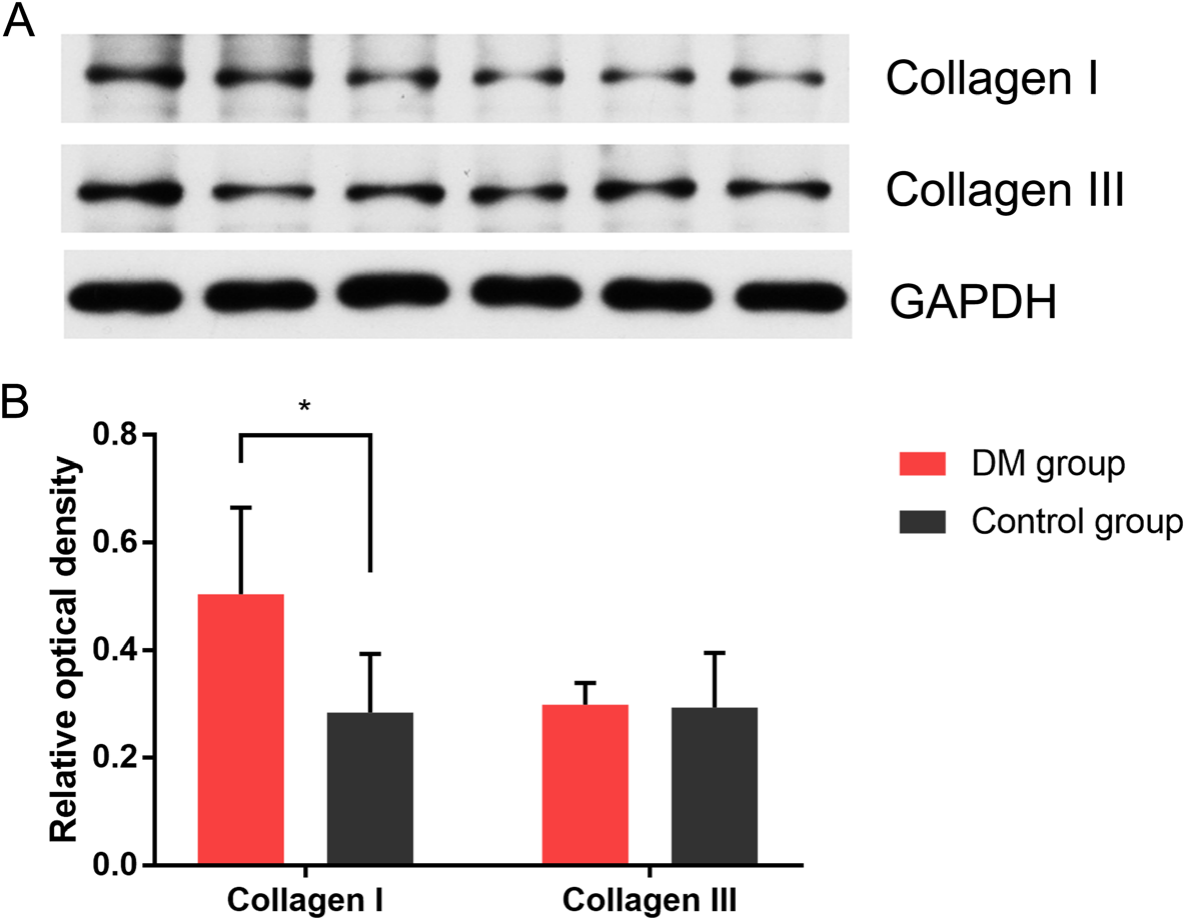
Western blotting and quantitative results of DM group (*n* = 40) and control group (*n* = 46). **a**) Western blotting results of representative sections. **b**) Relative optical density of western blotting results. * *P* < 0.001

Collagen I and Collagen III protein expression level were also determined by Western blotting, showed in Fig. 4. The optical density (divided by GAPDH) of Collagen I was higher in DM group than the control group (0.504 ± 0.161 vs. 0.297 ± 0.040, *P* < 0.001), while there was no difference of Collagen III (0.284 ± 0.109 vs. 0.293 ± 0.101, *P* = 0.685).

### Linear regression analysis

**Fig. 5:**
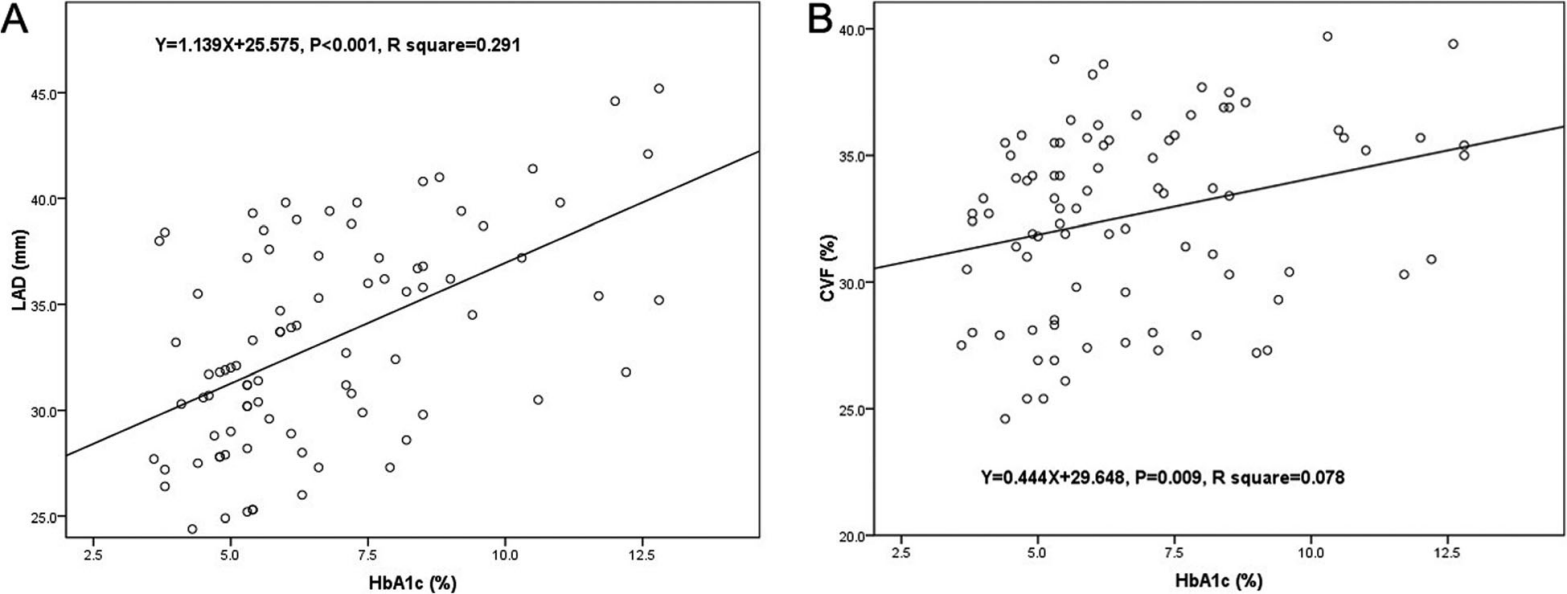
Univariate linear regression of HbA1c and LAD (**a**) and CVF (**b**). DM, diabetes mellitus; LAD, left atrium diameter

A univariate linear regression analysis was conducted to investigate the potential relationship between glycemic control and LAD, as well as CVF (Fig. 5), which showed that there was a significant linear relationship between LAD and HbA1c (Y = 1.139X + 25.575, *P* < 0.001, R^2^ = 0.291), and also there was a significant association between CVF and HbA1c (Y = 0.444X + 29.648, *P* = 0.009, R^2^ = 0.078). Multivariate linear regression analysis demonstrated that age and HbA1c were associated with LAD and CVF (*P* < 0.05), showed in Table 3.

**Table 3:** Multivariate linear regression analysis of LAD and CVF with common risk factors

Variables	LAD				CVF			
	B	BE	β	P	B	BE	β	P
Constant	8.614	4.541	–	0.061	25.621	4.173	–	< 0.001
Age	0.203	0.054	0.337	0.000	0.098	0.049	0.215	0.048
Sex	−0.054	0.926	−0.005	0.954	−0.139	0.848	−0.018	0.871
BMI	0.278	0.124	0.201	0.027	− 0.087	0.114	− 0.083	0.447
Smoking	1.061	0.989	0.093	0.287	−0.130	0.913	−0.015	0.887
Hypertension	−0.700	0.932	−0.066	0.455	0.436	0.862	0.054	0.614
HbA1c	0.942	0.188	0.446	0.000	0.449	0.170	0.282	0.010

BMI, Body mass index; HbA1c, hemoglobin A1c

## Discussion

In the present study, we compared the echocardiography results of the DM group and the control group at first, then we collected the precious atrium samples during the operation and analyzed the atrial fibrosis level. It was found that the atrium was significantly enlarged for the DM patients, and the atrial fibrosis was also obviously severe for DM patients, compared with the control group. Most importantly, a significant correlation was also confirmed between the glycemic control and atrial structural remodeling.

Atrium enlargement, manifested in the expansion of left atrium size, is represented by LA anteroposterior diameter or LA volume, which sometimes are indexed for body surface area or height [16]. Several cohort studies have been launched to investigate the relationship between DM status and LA diameter or LA volume. However, the results are inconsistent. On the one hand, some studies found that LAD or LAVI of DM patients were similar with controls, including CARDIA (Coronary Artery Risk Development in Young Adults) study and the TODAY (Treatment Options for Type 2 Diabetes in Adolescents and Youth) trial. CARDIA study enrolled 2903 young adults (age 23–35 years) and investigated the influence of some modifiable cardiovascular risk factors on LA size, and the results showed that diabetes was not associated with unindexed LA diameter and LA diameter indexed for body surface area or height [17]. Another relatively small-size cohort study TODAY trial enrolled 455 adolescents with type 2 diabetes, which demonstrated that LA diameter, did not correlate with HbA1c level [11]. In this study, no statistical difference of LAD was found between the DM group and the control group for the subgroup analysis of AF patients, which might be the results of insufficient sample size.

On the other hand, more pieces of evidence have indicated a confirmed association between LA enlargement and diabetes status or poor glycemic control [12, 18–20]. Our findings were compliable with the latter opinion. We not only found that patients with DM had a larger LA size, but also that the LAD was significantly correlated with HbA1c. These controversies could be attributed to different enrollment criteria or different LA measure methods. Notably, all of the patients enrolled in our study were complicated by CAD and ready to receive CABG operation, which might affect the results between LAD and DM status. Except for LAD and LAVI, we also found that IVST and E/A ratio were also significantly different between the two groups, which correlates well with Demir’s study [14]. The thickness of interventricular septum was increased, while the E/A ratio was decreased for DM patients, implicating an apparent diastolic dysfunction [12].

Atrial fibrosis has been proven to be a vital process of structural remodeling and commonly present in AF patients [21, 22]. Therefore, the examination of atrial fibrosis has become an essential step in determining the fundamental remodeling level [23]. Our previous studies also confirmed that AF patients had significantly severe fibrosis than SR patients, and this process was activated by TGF-beta1/alpha-SMA/Col I profibrotic pathway [24–26]. However, no previous reports compared the atrial fibrosis between DM patients and non-DM patients. In this study, we quantified the atrial fibrosis using an indicator CVF, which was calculated with different sections. The following linear regression analysis indicated a strong co-relationship for CVF and HbA1c, which give us a clue that the glycemic control might be influential for atrial fibrosis in DM patients. Although the link between atrial fibrosis and glycemic control has seldom been established before, other pieces of evidence reporting the association between fibrosis and high glucose level were published on other organs or cell lines [27–30]. Zhang et al. have reported that high glucose could induce cardiac fibrosis in a STZ-induced diabetic mice model, while the miR-155 regulated cardiac fibrosis via the TGF-beta1-Smad 2 signaling pathway [31]. Another study found that hyperglycemia could lead to atrial dilation and interstitial fibrosis, ionic remodeling, and increased vulnerability to AF [32]. However, the underlying mechanism about this association remains mostly unclear and requires more translational and basic studies in future. What’s more, we analyzed the fibrosis composition by western blot and found that Collagen I was more expressed in DM patients, while Collagen III not. This finding was also in compliance with our previous report (25), which demonstrated that Collagen I rather than Collagen III was the significant components of atrial fibrosis in AF patients.

Despite those robust findings, several limitations of this study must be noted. First, as we mentioned before, all patients enrolled in this study were complicated by CAD and given the CABG operation, which might lead to a systematic error for the final results. Second, the echocardiography was completed unblind, resulting in some subjective bias. Last but not least, the rice-size sample was acquired from the incision of the right atrium, small and local, which cannot reflect the whole situation of the heart. Recently, a non-invasive state-of-art imaging technique has emerged to investigate the atrial fibrosis, that is the late gadolinium-enhanced cardiac magnetic resonance (LGE-CMR), which has a superiority of non-invasive and accuracy on whole heart level [33]. Future research on atrial fibrosis may use this technique as a replacement for traditional sections or western blotting.

## Conclusions

In summary, we examined the atrial structural remodeling of DM patients and found that DM was associated with significant structural remodeling, including the atrium enlargement and atrial fibrosis, while the structural remodeling severity was linked to the glycemic control level.

## Data Availability

The datasets used and analyzed during the current study are available from the corresponding author on reasonable request.

## Abbreviations

AF: Atrial fibrillation
BMI: Body mass index
CABG: Coronary artery bypass graft
COPD: Chronic obstructive pulmonary disease
CVF: Collagen volume fraction
DM: Diabetes mellitus
FBG: Fast blood glucose
HbA1c: Hemoglobin A1c
IVST: Interventricular septum thickness
LAD: Left atrium diameter
LAVI: Left atrium volume index
LDL-C: Low-density lipoprotein cholesterol
LVEF: Left ventricular ejection fraction
NYHA: New York heart association
SR: Sinus rhythm
T2DM: Type 2 diabetes mellitus
TTE: Transthoracic echocardiography
